# Consumption of interesterified palm oil leads inflammation of white adipose tissue and triggers metabolic disturbances in mice on a high-fat diet

**DOI:** 10.1038/s41598-024-63488-9

**Published:** 2024-05-31

**Authors:** Bruna Cadete Martins, Mayara da Silva Ribeiro, Ananda Vitoria Silva Teixeira, Thamara Cherem Peixoto, Patrícia Cristina Lisboa, Fabiane Ferreira Martins, Vanessa Souza-Mello, Julio Beltrame Daleprane

**Affiliations:** 1 Laboratory for Studies of Interactions Between Nutrition and Genetics, LEING, Department of Basic and Experimental Nutrition, Rio de Janeiro State University, Rio de Janeiro, Brazil; 2 Department of Morphology, Federal University of Rio Grande Do Norte, Rio Grande do Norte, Brazil; 3 Laboratory of Endocrine Physiology, Department of Physiological Sciences, State University of Rio de Janeiro, Rio de Janeiro, RJ 20551-030 Brazil; 4 Laboratory of Morphometry, Metabolism and Cardiovascular Diseases, Biomedical Center, Institute of Biology, Rio de Janeiro State University, Rio de Janeiro, Brazil

**Keywords:** Obesity, Interesterified fat, Insulin resistance, Hepatic steatosis, Biochemistry, Physiology, Medical research

## Abstract

Growing obesity is linked to shifts in dietary patterns, particularly the increased intake of ultra-processed high-fat foods. This study aimed to evaluate the effects of interesterified palm oil consumption on glucose homeostasis, adipose tissue remodeling, and hepatic lipogenesis in C57BL/6 mice fed a high-fat diet. Sixty C57BL/6 mice were divided into four groups (n = 15): the control group (C) fed a standard diet (4% soybean oil), the high-fat group (HF) (23.8% lard), the high palm oil fat group (HFP) (23.8% palm oil), and the high interesterified palm fat group (HFI) (23.8% interesterified palm oil) for 8 weeks (all groups received 50% energy from lipids). The HFI group exhibited higher body mass than the HF group (+ 11%, *P* < 0.05), which was attributed to an increased percentage of fat mass. Plasma concentrations of IL-6, insulin, and HOMA-IR were also elevated in the HFI group. Both the HFP and HFI groups showed hypertrophied adipocytes and pancreatic islets, increased alpha and beta cell masses, hepatic steatosis, low expression of genes related to beta-oxidation, and upregulated lipogenesis. In conclusion, the consumption of interesterified palm oil alters inflammatory and glucose profiles.

## Introduction

For years, studies have highlighted numerous health hazards associated with trans-fat consumption^1–4^. In response to the ban on trans fats, a range of substitutes has been developed, including interesterified fats, palm oil, protein-based replacements, carbohydrate-based alternatives, synthetic fats, and structured oleogels^5–7^. Palm oil (PO) is the primary vegetable fat used to replace trans fat because of its semisolid consistency and ease of use in bakery products^8^. It is the cheapest basic oil and is produced entirely without genetic modifications and solvent extraction^9^.

Unlike hydrogenation, interesterification does not affect the degree of fat saturation or cause isomerization of fatty acids (FA); therefore, interesterified fat has the same FA profile as the raw material used in its production^10^. Interesterification involves the redistribution of FAs on the glycerol skeleton, typically randomly altering the fat's melting properties^11,12^. This process increases desaturated and trisaturated triacylglycerol (TAG) levels by introducing saturated fatty acids (palmitic or stearic) at the sn-2 position of glycerol^13–15^. FAs occupying the sn-2 position have better bioavailability than those occupying other positions in glycerol and thus are better absorbed in the body^16^.

Although the effects of palm oil consumption still need to be clarified, studies have reported that its consumption reduces low-density lipoprotein (LDL) and plasma TAG^17,18^, predisposes offspring to obesity in adulthood^19^ and negatively affects food intake regulation during pregnancy and lactation^20^. Palm oil is also associated with increased plasma TAG, endothelial dysfunction^21^, weight gain, insulin resistance (IR), and hyperglycemia^22^. However, little is known about the effects of consuming interesterified fat on human health. It is believed that the sn-2 position has better availability and is more easily absorbed by the body^15^. The oil after being modified by the interesterification process would have more deleterious effects owing to the better use of saturated fatty acids. Moreover, it is optional for food labels to inform consumers about the presence of interesterified fat, which makes it difficult for consumers to know how much of it are they consuming.

The modification of dietary patterns, especially through the consumption of highly processed foods rich in saturated and interesterified fats and simple sugars, along with reduced physical activity, results in an increased calorie expenditure that leads to an energy imbalance and systemic changes that promote increased body mass and adiposity^23–25^. In addition to changes in adipose tissue, body weight gain has negative consequences in other non-adipose tissues such as the skeletal muscle, heart, liver, and pancreas and may cause abnormal fat accumulation in these tissues, overexpression of inflammatory cytokines, impaired function, and insulin resistance^26^. The present study aimed to evaluate the effects of interesterified palm oil on glucose homeostasis, adipose tissue remodeling, and hepatic lipogenesis in C57BL/6 mice fed a high-fat diet.

## Results

### Characterization of dietary fats—palm oil and interesterified palm oil

The fatty acid profiles of soybean oil (SO), palm oil (PO), and Interesterified palm oil (IPO) contain approximately 22% saturated fatty acids (SFAs) for SO and 52% for OP and IPO (Table 1). As expected, unsaturated fatty acid (UFAs) levels were higher in OS (78%) than in OP (46%) and IOP (48%). Polyunsaturated fatty acids (PUFAs) represented 49% of the total UFAs in SO, whereas monounsaturated fatty acids (MUFAs) represented higher proportions of UFAs in OP (41%) and IOP (41%). Linolelaidic acid (49%) and oleic acid (25%) were more abundant in SO, whereas palmitic and oleic acid were more abundant in OP (43% and 39%, respectively) and IOP (39% and 39%, respectively). IPO presents fatty acid profiles similar to those of PO. Therefore, using PO and IPO diets, only the differential TAG composition (fatty acid distribution) was assessed, thus avoiding other variables and interference factors, such as the fatty acid profile and the relative proportion of saturated, monounsaturated, and polyunsaturated fatty acids.

**Table 1:** Fatty acid composition of soybean oil (SO), palm oil (PO) and interesterified palm oil (IPO).

Fatty acid		SO (%)	PO (%)	IPO (%)
C12:0	(Lauric acid)	0.22	1.26	3.24
C13:0	(Tridecanoic acid)	0.00	0.05	0.00
C14:0	(Myristic acid)	0.26	1.73	1.95
C15:0	(Pentadecanoic acid)	0.05	0.12	0.11
C16:1	(Palmitoleic acid)	0.18	0.25	0.22
C16:0	(Palmitic acid)	13.50	43.92	39.33
C17:1	(cis-10-heptadecenoic acid)	0.15	0.06	0.00
C17:0	(Margaric acid)	0.13	0.16	0.13
C18:2n6c	(Linolelaidic acid)	49.46	5.18	7.08
C18:1n9c	(Oleic acid)	25.76	39.50	39.14
C18:1n9t	(Elaeic acid)	2.41	1.09	1.90
C18:0	(Stearic acid)	4.38	5.56	5.22
C20:2	(Cis-11,14-eicosadienoic acid)	0.07	0.03	0.00
C20:1n9	(Cis-11-Eicosenoic acid)	0.24	0.11	0.18
C20:0	(Arachidic acid)	0.65	0.55	0.71
C22:0	(Behenic acid)	1.40	0.17	0.26
C23:0	(Tricosanoic acid)	0.21	0.02	0.07
C24:0	(Lignoceric acid)	0.96	0.23	0.46
Total (%)		100	100	100
Saturated fatty acids		21.7	53.8	51.5
Monoinsaturated fatty acids		28.74	41.01	41.44
Polyunsaturated fatty acids		49.52	5.22	7.07
Total (%)		100	100	100

SO, Soybean oil group; PO, palm oil group; IPO, interesterified palm oil group.

The triacylglycerol (TAG) composition in SO, PO, and IPO was evaluated and presented as percentages (Table 2). Soybean oil exhibited high LLL (21.78%) and OLL (25.75%) concentrations, whereas palm oil exhibited elevated levels of POP (31.57%) and POO (24.86%). Interesterified palm oil showed POP (32.46%) and POO (25.46%) values similar to those of palm oil. Soybean oil had a lower percentage of DAG (3.7%) than palm oil (6.2%) and interesterified palm oil (6.82%). The sum of monoacylglycerol (MAG) and free fatty acids (FFA) was higher in interesterified palm oil (0.97%) than in soybean oil (0.9%) and palm oil (0.82%).

**Table 2:** Triacylglycerol composition and relative composition of triacylglycerol, diacylglycerol, monoacylglycerol and free fat acids of soybean, (SO), palm (PO) and interesterified palm oil (IPO).

Triacylglycerol (%)	SO	PO	IPO
MPP	0.19	0.46	0.54
LaOP	0.22	0.13	0.10
PPP	0.48	8.71	8.30
LLL	21.78	0.57	0.78
MOP	0.21	2.01	1.97
MLP	0.39	0.14	0.27
PPS	0.91	1.87	1.92
POP	2.96	31.57	32.46
PLP	1.36	5.59	5.98
POS	3.45	7.00	5.98
POO	4.15	24.86	25.46
POL	4.00	7.56	8.20
PLL	10.12	0.52	0.64
SOO	6.87	1.88	1.24
OOO	7.91	2.82	2.17
OLO	8.35	0.99	0.84
OLL	25.75	2.40	2.23
NI	0.90	0.93	0.88
Total (%)	100	100	100
MAG + FFA	0.9	0.82	0.97
DAG	3.7	6.2	6.82
TAG	95.4	92.98	92.21
Total (%)	100	100	100

SO, Soybean oil group; PO, palm oil group; IPO, interesterified palm oil group.

The regiospecific distributions of fatty acids at the sn-1,3 and sn-2 positions in the TAGs SO, PO, and IPO are shown in Table 3. We analyzed the regiospecific distribution of fatty acids in TAGs of SO, PO, and IPO, focusing on the sn-1,3 and sn-2 positions. We found a distinct distribution pattern of saturated and unsaturated fatty acids across all analyzed oils. At the sn-1,3 positions, SO exhibited a higher concentration of unsaturated fatty acids (71.1%) than saturated fatty acids (28.9%). In contrast, PO showed a predominance of saturated fatty acids (74.9%) over unsaturated fatty acids (25.1%). After the interesterification process, IPO demonstrated a composition similar to that of SO, with a higher proportion of unsaturated fatty acids (76.9%) than of saturated fatty acids (23.1%). In contrast, at the sn-2 position, the distribution pattern shifted significantly, particularly for IPO. SO maintained a high level of unsaturated fatty acids (98.6%) and minimal levels of saturated fatty acids (1.4%). PO had a more balanced distribution, with 17.3% saturated fatty acids and 82.7% unsaturated fatty acids. Remarkably, after interesterification, IPO exhibited a dramatic increase in saturated fatty acids (70.9%) and a decrease in unsaturated fatty acids (29.1%). These results underscore the significant impact of the interesterification process on fatty acid distribution within TAGs, particularly at the sn-2 position, which could influence the nutritional and physical properties of the oils.

**Table 3:** The specific distribution of fatty acids in the sn-1.3 and sn-2 positions of triacylglycerols present in soybean oil (SO). palm oil (PO) and interesterified palm oil (IPO).

Position of fatty acid	SO (%)	PO (%)	IPO (%)
Saturated fatty acid *sn*-1.3	28.9	74.9	23.1
Unsaturated fatty acid *sn*-1.3	71.1	25.1	76.9
Total (%)	100	100	100
Saturated fatty acid *sn*-2	1.4	17.3	70.9
Unsaturated fatty acid *sn*-2	98.6	82.7	29.1
Total (%)	100	100	100

SO, Soybean oil group; PO, palm oil group; IPO, interesterified palm oil group.

### Interesterified palm oil promotes greater body weight gain and fat mass than palm oil

**Figure 1:**
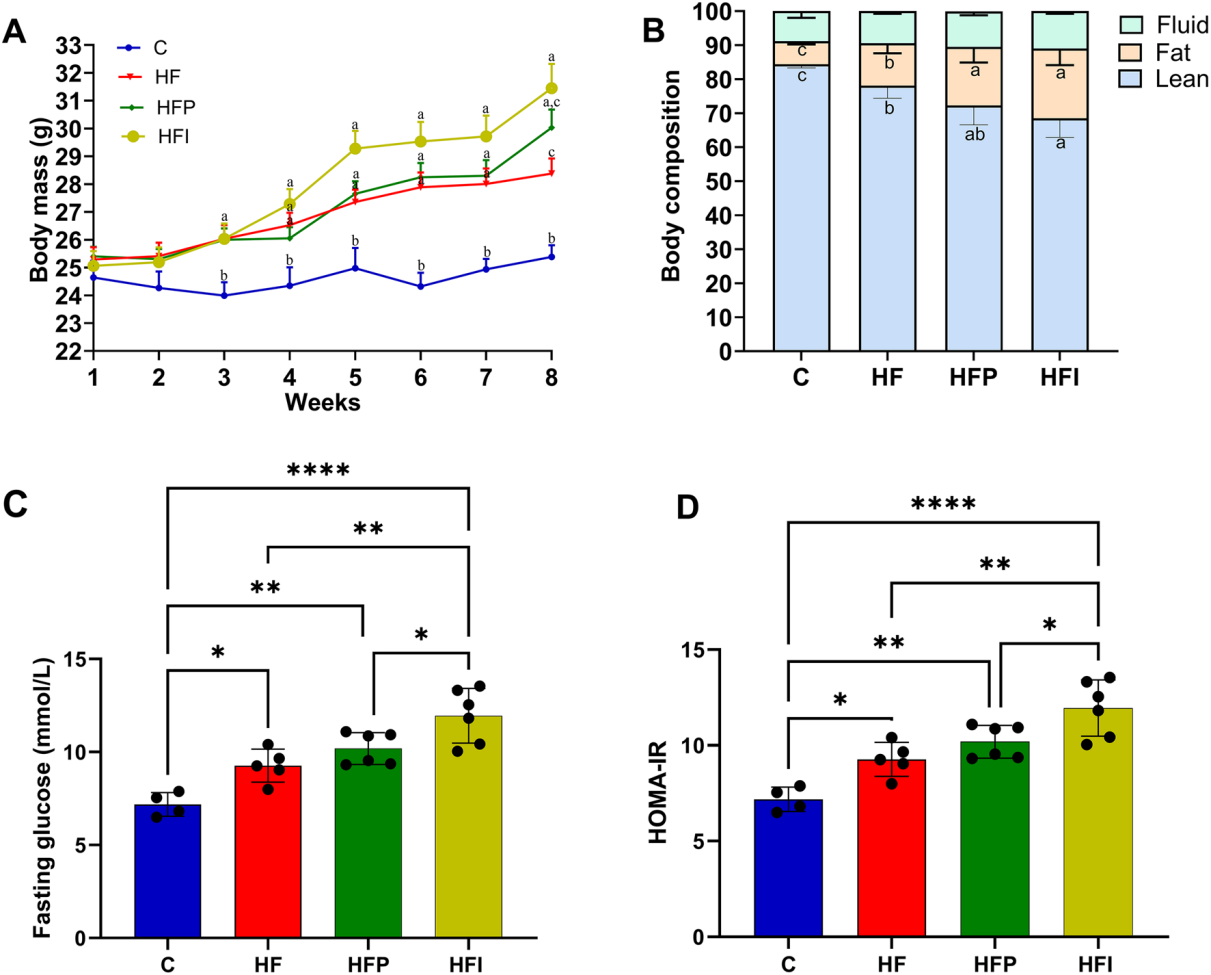
The consumption of interesterified palm oil promoted greater body weight gain, fat mass, and worse effects on glucose metabolism. (**A**) Experimental model (**B**) Weekly body mass evolution. (**B**) Body composition. One-way ANOVA followed by Tukey's post-hoc test (mean ± SD, n = 5). Different letters indicate significant differences between groups (*P* < 0.05). *P < 0.05, **P < 0.01, ****P < 0.0001 for the remaining parameters. C, Control; HF, high-fat diet; HFP, high-fat diet with palm oil; HFI, high-fat diet with interesterified palm oil.

Body mass was not significantly different between mice in different groups in the second week, but from the third week onwards, the groups consuming a high-fat diet showed significantly greater body mass gain compared to the control group (Fig. 1A). At eight weeks, the HFI group had a higher body mass than the HF group (+ 11%, *P* < 0.05), whereas the HFP group had an intermediate body mass. The increase in body mass observed in both the HFI and HFP groups was due to the higher percentage of body fat (Fig. 1B). The average feed consumption was similar among the groups throughout the experimental period (eight weeks) (Fig. 1C). This scenario remained the same for the duration of the experiment (eight weeks). Among the three diets, the consumption of interesterified palm oil promoted greater body weight gain and fat mass.

### Biochemical analysis of plasma from experimental mice

Notably, for both triglycerides and leptin, the levels observed in the HFP group were not significantly different from those observed in the HF or HFI groups, whereas the levels between the HF and HFI groups were significantly different (*P* < 0.05). Plasma cholesterol, AST, ALT, and IL-1 were higher in the HFP and HFI groups than in the HF group (*P* < 0.05). All groups that consumed a high-fat diet showed equal levels of plasma adiponectin, TNF-a, and GIP. The HFI group showed higher plasma IL-6 levels than the other groups (Table 4, *P* < 0.05). Plasma insulin and GLP-1 levels were highest in the HFI group, followed by the HPF and HF groups; group C showed the lowest levels (*P* < 0.05) (Table 4).

**Table 4:** Biochemical analysis of plasma from animals submitted to the experimental protocol.

Biochemistry	C	HF	HFP	HFI
Triglyceride (mg/dL)	63.6 ± 7.6^c^	110.8 ± 11.8^b^	137.8 ± 21.4^a.b^	168.9 ± 31.5^a^
Cholesterol (mg/dL)	78.8 ± 9.0^c^	203.3 ± 9.1^b^	244.3 ± 25.2^a^	252.9 ± 17.2^a^
AST (UL/L)	24 ± 2.3^c^	36 ± 3.4^b^	42 ± 2.9^a^	42 ± 2.9^a^
ALT (UL/L)	12 ± 2.7^c^	25 ± 3.4^b^	36 ± 4.7^a^	37 ± 1.5^a^
Leptin (ng/ml)	5.6 ± 0.9^c^	12.4 ± 2.0^b^	14.5 ± 1.0^a.b^	15.1 ± 1.3^a^
Adiponectin (ng/ml)	101.2 ± 2.7^b^	62.9 ± 4.8^a^	55.4 ± 8.6^a^	53.6 ± 2.5^a^
Fasting glucose (mg/dL)	7.1 ± 0.9^d^	9.2 ± 1.2^c^	10.1 ± 1.7^b^	11.9 ± 1.3^a^
Insulin (μUI/L)	43.95 ± 2.7^d^	70.25 ± 1.6^c^	77.35 ± 3.2^b^	87.75 ± 3.9^a^
HOMA-IR	14.5 ± 2.7^d^	28.0 ± 3.5^c^	34.9 ± 3.9^b^	47.5 ± 3.8^a^
GIP (pg/ml)	234.3 ± 15^b^	194.9 ± 4.9^a^	171.8 ± 12.8^a^	182.65 ± 16^a^
GLP-1 (pg/ml)	42.3 ± 2.6^c^	12.95 ± 3.9^b^	9.35 ± 1.8^a.b^	8.15 ± 1.0^a^
TNFα (pg/ml)	20.9 ± 3.4^b^	100.5 ± 9.1^a^	110.1 ± 3.6^a^	122 ± 14.0^a^
IL-6 (pg/ml)	13.4 ± 3.4^c^	51.6 ± 7.2^b^	62.7 ± 4.0^b^	74.8 ± 6.6^a^
IL-1β (pg/ml)	62.7 ± 7.7^c^	586.1 ± 202.0^b^	1116.0 ± 260.2^a^	1311.1 ± 145.8^a^

One-way ANOVA followed by Tukey's post-test (mean ± SD. n = 5). Different letters indicate a significant difference between the groups (*P* < 0.05). C, Control; HF, high-fat diet; HFP, high-fat diet with pal oil; HFI, high-fat diet with interesterified palm oil.

### Palm oil and interesterified palm oil intake increases hypertrophy and lipid peroxidation in epididymal white adipose tissue (eWAT)

**Figure 2:**
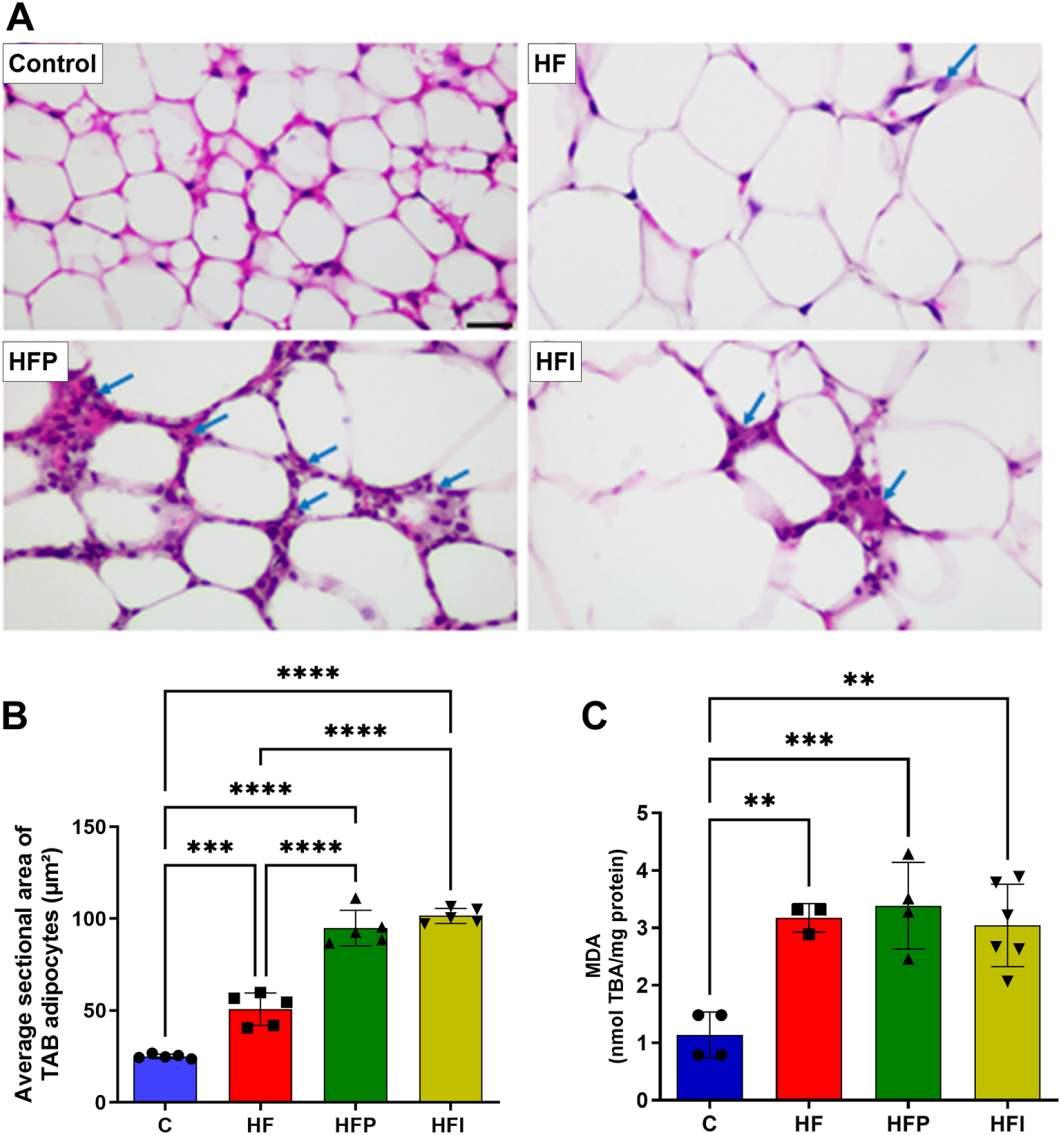
The consumption of palm oil and interesterified palm oil increased hypertrophy, inflammation (arrows), and lipid peroxidation in eWAT. (**A**) eWAT histology, (**B**) average cross-sectional area of white adipocytes, and (**C**) MDA. Histological images captured using a 20X objective, calibration bar = 50 μm. One-way ANOVA followed by Tukey’s post-hoc test (mean ± SD, n = 5). Significant differences are indicated by *P < 0.05, **P < 0.01, ***P < 0.001 and ****P < 0,0001. C, Control; HF, high-fat diet; HFP, high-fat diet with palm oil; HFI, high-fat diet with interesterified palm oil.

Histological images were obtained to evaluate the morphology of eWAT. Photomicrographs depicted smaller adipocytes in group C, whereas hypertrophied adipocytes in the HF, HFP, and HFI groups (Fig. 2A). In addition to hypertrophy, inflammatory infiltrates were observed (indicated by blue arrows). The mean adipocyte cross-sectional areas in the HFP and HFI groups were similar and greater (+ 46.5% and + 50%, respectively) than those in the HF group (*P* < 0.0001) (Fig. 2B). The levels of thiobarbituric acid reactive substances (TBARS) were higher and similar between the HF and HFP groups (+ 32% and + 49%, respectively) than in the HFI group (*P* < 0.01) (Fig. 2C). Ingestion of palm oil and interesterified palm oil increased hypertrophy and lipid peroxidation in eWAT.

### High-fat diet leads to the overexpression of inflammation-related genes in eWAT

**Figure 3:**
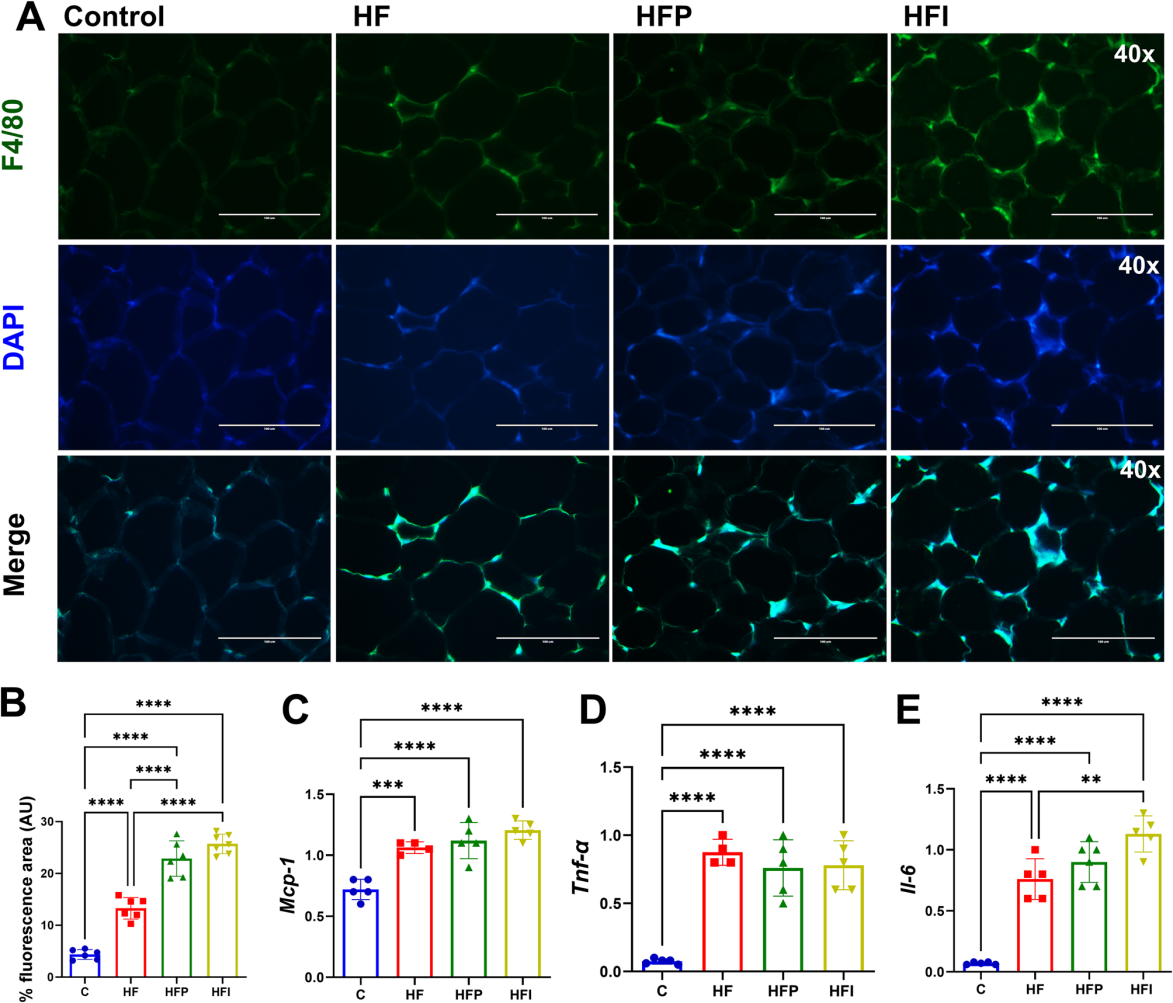
High-fat diet increases the expression of genes related to inflammation in eWAT. (**A**) F4/80 immunofluorescence in eWAT, (**B**) MPC-1 expression, (**C**) TNF-αexpression, and (**D**) IL-6 expression. One-way ANOVA followed by Tukey’s post-hoc test (mean ± SD, n = 5). Significant differences are indicated by *P < 0.05, **P < 0.01, ***P < 0.001 and ****P < 0.0001. C, Control; HF, high-fat diet; HFP, high-fat diet with palm oil; HFI, high-fat diet with interesterified palm oil.

To evaluate the expression of inflammation-related genes and confirm that inflammatory infiltration is associated with adipocyte hypertrophy, we performed immunofluorescence for F4/80 and evaluated the gene expression of inflammatory markers in eWAT. The intensity of F4/80 immunofluorescence was stronger in the HFI group than in the HF group, and that of the HFP group was intermediate between that of the two groups (Fig. 3A,B). The expression of Mcp-1 (Fig. 3C) and Tnf-α (Fig. 3D) was also equal and higher in the HF, HFP, and HFI groups than in the controls (Mcp-1:14 times; Tnf-α 13 times, *P* < 0.001). However, the expression of Il-6 was higher in the HFI than in the HF group (Fig. 3E, + 36%, *P* < 0.01). This change in Il-6 level may be because it is the most sensitive to the cytokine storm; it takes longer (< 8 weeks) for other interleukins to increase in response to the inflammation.

### Palm oil stimulates lipogenesis and reduces beta-oxidation

**Figure 4:**
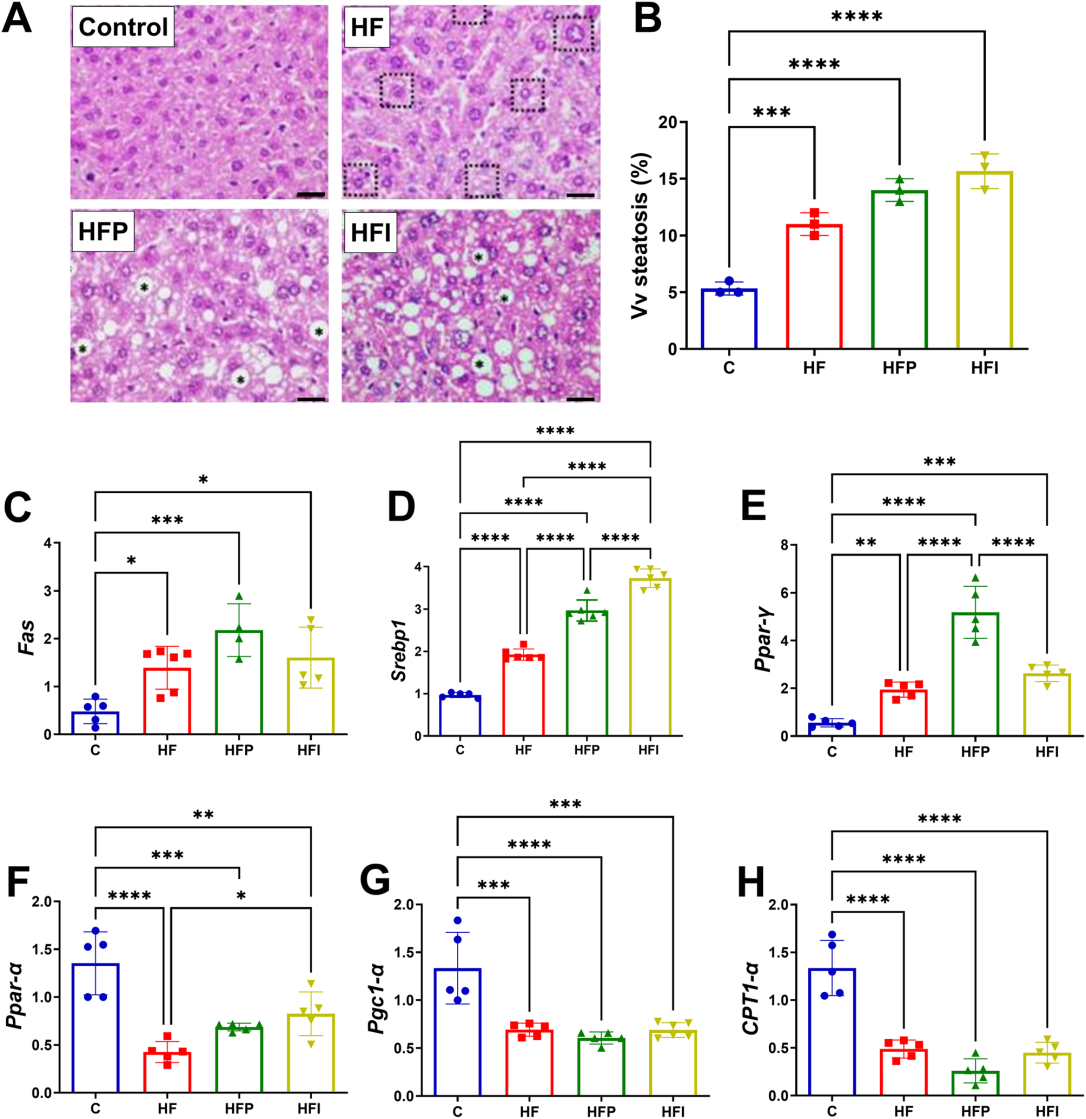
The consumption of palm oil and interesterified palm oil indicated hepatic steatosis. (**A**) Liver morphology H&E; dotted square represents hepatic glycogen degradation, * indicates macrosteatosis, (**B**) Vv steatosis (%), (**C**), (**D**), (**E**) expression of genes related to lipogenesis, (**F**), (**G**), (**H**) expression of genes related to beta-oxidation. One-way ANOVA followed by Tukey’s post-hoc test (mean ± SD, n = 5). Significant differences are indicated by *P < 0.05, **P < 0.01, ***P < 0.001 and ****P < 0.0001. C, Control; HF, high-fat diet; HFP, high-fat diet with palm oil; HFI, high-fat diet with interesterified palm oil.

To assess the potential correlation between the deleterious effects of interesterified palm oil in eWAT and the liver, hepatic steatosis and the expression of genes involved in lipogenesis and beta-oxidation were evaluated. More micro- and macrolipid inclusions were observed in the cytoplasm of hepatocytes (Fig. 4A), as well as, a higher fraction of steatosis volume (Vv) (HF: + 107%, HFP: + 287%; HFI: + 314%, *P* < 0.01; Fig. 4B) in the groups that consumed a high-fat diet. The expression of the lipogenic *Fas* gene was higher and similar among all high-fat groups (Fig. 4C). *Srebp-1* was overexpressed in the HFP group than in the HF and HFI groups ((Fig. 4D, + 39% and + 59%, respectively, *P* < 0.0001). *Pparγ* was higher in the HFP group than in the HF and HFI groups (Fig. 4E, + 40% and 37%, respectively, *P* < 0.0001). *Pparα* was overexpressed in the HFI than in the HF group (Fig. 4F, + 52%, *P* < 0.0001), and expression levels in the HFP group were intermediate between that of the two groups. The expression levels of *Pgc1-α* and *Cpt1-α* were lower and similar among the three high-fat groups (Fig. 4G,H). These findings suggest that interesterified palm oil intake leads to greater lipogenic activity and lower beta-oxidation, which may contribute to lipid accumulation.

### Palm oil and interesterified palm oil lead to pancreatic hypertrophy and increased alpha and beta cell mass

**Figure 5:**
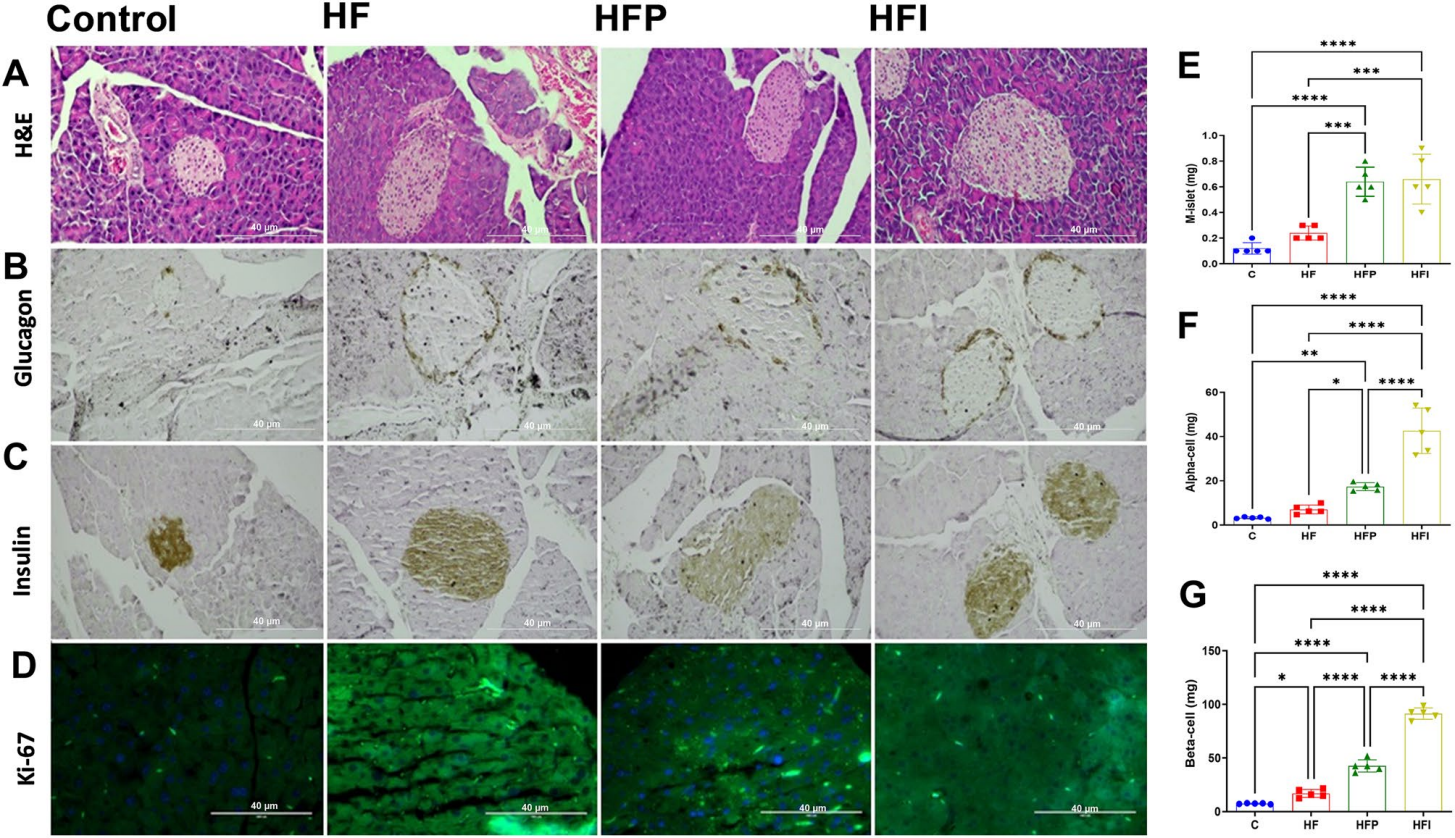
Palm oil and interesterified palm caused pancreatic hypertrophy and increased alpha and beta cell mass. (**A**) Pancreatic morphology-HE. (**B**) Alpha-cell labeled with anti-glucagon, (**C**) Beta-cell labeled with anti-insulin. (**D**) Immunofluorescence of Ki-67. (**E**) Islet mass—M [islet, pancreas], (**F**) alpha-cell mass [Vv [alpha-cell], and (**G**) beta-cell mass [Vv [beta-cell]. One-way ANOVA followed by Tukey's post-hoc test (mean ± SD, n = 5). Significant differences are indicated by *P < 0.05, **P < 0.01, ***P < 0.001 and ****P < 0.0001. C, Control; HF, high-fat diet; HFP, high-fat diet with palm oil; HFI, high-fat diet with interesterified palm oil.

Fasting blood glucose levels were higher in the HFI than in all high-fat diet groups (*P* < 0.05) (Table 4). HOMA-IR was higher in the HFP group than in the HFI (+ 26.5, *P* < 0.05) and the HF groups (+ 41%, *P* < 0.01) (Fig. 1D). The islets of the groups that consumed a high-fat diet were hypertrophied compared with those of group C (Fig. 5A,E). To better understand pancreatic changes, the masses of alpha and beta cells were measured to assess glycemic homeostasis. The masses of alpha and beta cells were higher in the HFI group than in the HFP and HF groups (+ 166% and + 175%, respectively; *P* < 0.0001) (Fig. 5B,F). The same was observed for beta cell mass (+ 151% and + 438%, respectively; *P* < 0.0001) (Fig. 5C,G). This suggests that hypertrophy occurred due to an increase in pancreatic mass, probably due to greater pancreatic effort to maintain blood glucose levels. This increase was due to greater cellular anemia, as evidenced by the greater immunodensity of Ki-67 in the high-fat diet groups (Fig. 5D).

## Discussion

In this study, we assessed the effects of the consumption of unmodified and interesterified palm oil in a murine model of diet-induced obesity. We hypothesized that consuming interesterified palm oil along with a high-fat diet would be more damaging than consuming unmodified palm oil alone. However, we found that the adverse effects of the high-fat diet overshadowed any variances in lipid sources.

Consumption of palm oil and interesterified palm oil induced adipocyte hypertrophy with a larger average sectional area, increased plasma leptin, F4-80 immunoreactivity, and gene expression of inflammatory markers. However, *Il-6* expression increased with the consumption of interesterified palm oil. Additionally, we observed micro- and macrovesicular lipid inclusions within the hepatocytes of HFI, with elevated steatosis volume, plasma TAG and cholesterol concentrations, and decreased expression of beta-oxidation-related genes (*Ppargama*, *Pgc1a*, and *Cpt1a*). Among genes involved in lipogenesis, *Srebp-1* expression was higher in the palm oil consumption group, and *Ppara* and *Fas* expression was similar between the oil-receiving groups.

Excess weight is characterized by hypertrophy and hyperplasia of adipose tissue, which triggers a local inflammatory response and activates immune cells^27^. Adipose tissues contain multiple immune cells that monitor and maintain the integrity and hormonal sensitivity of adipocytes. The morphological changes caused by excess weight increase the total number of macrophages, which is largely due to the recruitment of M1 polarized macrophages, which have a pro-inflammatory phenotype and secrete cytokines such as TNF-α. The increase in the number of macrophages and subsequently, in the ratio of M1 to M2 macrophages is a hallmark of adipose tissue inflammation that is consistent with obesity and is associated with the development of insulin resistance and metabolic diseases^28^. Increased FFAs following a high-fat diet can promote inflammation by indirectly binding to TLR4 and TLR2 through the adapter protein fetuin-A, resulting in the activation of NF-κB and JNK1 pathways. Once activated, these pathways can increase the synthesis and secretion of chemokines, such as the MCP-1 protein, from adipocytes or hepatocytes, leading to the infiltration of pro-inflammatory macrophages^29^. The consumption of palm oil and interesterified palm oil led to a similar increase in the plasma levels of the pro-inflammatory cytokines TNF-α and IL-1β, however, the consumption of palm oil led to higher plasma levels of IL-6, which demonstrates greater circulation of pro-inflammatory cytokines. The expression of inflammatory cytokines in white adipose tissue was also evaluated; MCP-1 and TNF-α levels were similar in all groups that consumed a high-fat diet and the expression of IL-6 was higher in the interesterified palm oil group than in the other high-fat diet groups. IL-6 is associated with proinflammatory effects in obesity that can induce insulin resistance^30,31^. This association may be responsible for the more pronounced RI observed in the interesterified PO-treated group.

These changes in adipose tissue reflect higher plasma leptin and lower plasma adiponectin levels, with metabolic repercussions in experimental animals. In addition to storing energy, the white adipose tissue is an endocrine organ that functions through adipokines, notably leptin and adiponectin. Leptin is secreted in proportion to the amount of WAT and regulates satiety in the central nervous system, thereby suppressing appetite and increasing energy expenditure. However, leptin resistance occurs when excess leptin circulates. While adiponectin secretion is inversely proportional to the amount of WAT, it improves insulin sensitivity in the skeletal muscle (glucose uptake and lipid oxidation), reduces gluconeogenesis and the accumulation of triglycerides in the liver, and induces cardiovascular protection^32,33^. The leptin to adiponectin ratio (L:A) correlates well with other aspects of metabolic dysregulation and the risk of chronic metabolic disease^34–36^. Individuals with obesity and a high L:A ratio tend to have delayed TAG clearance, as well as insulin and leptin resistance, making the L:A ratio a useful marker for metabolic syndrome^37^.

Obesity-related lipotoxicity contributes to peripheral insulin resistance^38^, leading to ectopic lipid accumulation^39^. Compared to palm oil consumption, the consumption of interesterified palm oil led to weight gain due to increased fat mass, hypertrophied adipocytes, higher fasting glycemia, serum insulin, and high HOMA-IR index, indicating that consumption of a high-fat diet with interesterified palm oil can cause deleterious effects on glucose metabolism due to insulin resistance. In insulin resistance, beta cells increase insulin secretion to compensate for the hypertrophy of the pancreas and an increase in mass. However, this compensatory response eventually fails, leading to the progressive decline of beta cell function^40^. Impaired insulin action results in defective regulation and increased rates of hepatic gluconeogenesis, which is considered the main mechanism leading to fasting hyperglycemia in type 2 diabetes mellitus^41^.

Ectopic lipid accumulation results in pancreatic steatosis owing to excess consumed energy^42^. Under these conditions, the pancreatic volume increases owing to excessive fat deposition. This is largely attributed to triglycerides and FFAs, while cytokine secretion increases^43,44^. Pancreatic steatosis is accompanied by islet hypertrophy and increased beta cell mass^43,45^ and can compromise normal pancreatic function^46,47^, leading to the development of exocrine and endocrine insufficiency^48^. Intesterified palm oil consumption appears to be more aggressive in pancreatic lipid infiltration, resulting in more deleterious effects. A higher concentration of plasma insulin may have occurred because of hypertrophy of pancreatic beta cells and lower plasma levels of incretins.

Excessive expansion of adipocytes can have detrimental health consequences, as excess free fatty acids enter other tissues and cause ectopic fat deposition by resynthesizing triglycerides. Sterol regulatory element binding protein (SREBP)-1c is a transcription factor of fatty acid synthase (FAS) and acetyl-CoA carboxylase (ACC), which promote the synthesis of cholesterol and triglycerides in the liver. SREBP-1c mRNA and protein levels are elevated in the liver under high-fat diet conditions^49^. In this study, we found a similar pattern of high gene expression of SREBP-1 as well as increased gene expression of FAS in the livers of experimental animals, which can be correlated with higher rates of lipogenesis and, consequently, greater lipid accumulation. The activation of SREBP can be used as an indicator of lipogenesis in the cell^49^. Higher levels of triacylglycerol, plasma cholesterol, and TAG were also consistent with the consumption of palm oil and interesterified palm oil, reinforcing the activation of the lipogenic pathway. SREBP-1 is the main protein associated with hypertriglyceridemia, and some studies have suggested its relationship with hypercholesterolemia^50^. In addition, it regulates lipogenesis in the liver, kidneys, skeletal muscle, and pancreas^51^. The lower expression of genes involved in beta-oxidation, PPARα, PCG1α, and CPT1-αwhich may reflect the dyslipidemia of animals in the high-fat diet groups, with the effects being particularly pronounced in the HFP and HFI groups.

TAGs are partially hydrolyzed in the stomach by gastric lipase, which preferentially hydrolyzes the sn-3 ester bond leading to the formation of sn-1, 2-diacylglycerols (DAGs), and FFAs. In the duodenum, the products of gastric digestion undergo lipolysis by various pancreatic lipases, which preferentially hydrolyze sn-1 and sn-3 bonds, releasing sn-2 monoacylglycerols (MAGs) and FFAs^52^. MAGs form mixed micelles with bile salts that are efficiently absorbed by passive diffusion. Intestinal fatty acid-binding proteins bind monounsaturated fatty acids more efficiently than saturated fatty acids and palmitic acid more efficiently than stearic acid^53^. Given this difference in absorption, we hypothesized that the consumption of interesterified palm oil in association with a high-fat diet would be more damaging than the consumption of unmodified palm oil. However, we observed that the adverse effects of the high-fat diet overshadowed lipid source differences. The consumption of interesterified palm oil in a normolipidemic and normocaloric diet model (AIN-93) resulted in metabolic effects similar to those of a high-fat diet with or without interesterified oil. This reinforces the superiority of the metabolic effects of a high-fat diet.

Owing to alterations in the fatty acid composition of the glycerol molecule, interesterification leads to an increased absorption of saturated fatty acids. Specifically, at the sn-2 position, where unsaturated fatty acids naturally reside, interesterification substitutes unsaturated fatty acids with saturated fatty acids. This heightened intake of saturated fatty acids can influence inflammatory responses in metabolically active tissues, including the liver, adipose tissue, muscles, and hypothalamus. The activation of inflammatory responses through innate immune receptors and cytokines is a hallmark of chronic low-grade inflammation and is frequently observed in overweight and obese individuals. Moreover, dietary lipids can modulate the phospholipid composition, particularly affecting skeletal muscle and cell membranes, thereby potentially affecting insulin sensitivity and glucose uptake. In this context, we propose that the primary contributors to impaired insulin signaling in the liver of the interesterified group are increased local inflammation and lipid accumulation.

**Figure 6:**
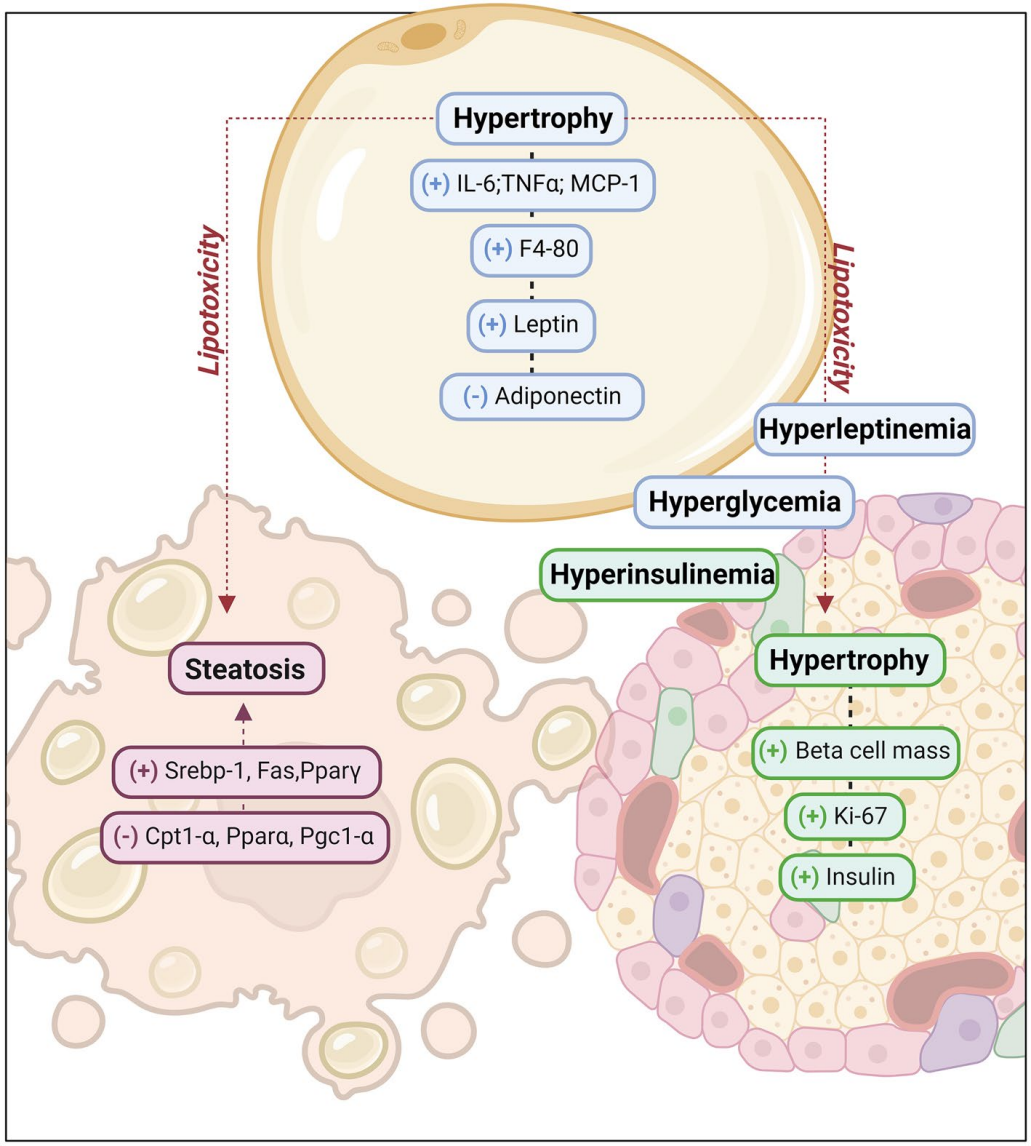
Palm oil and interesterified palm oil in a high-fat diet model caused morphofunctional changes in white adipose tissue. Hypertrophy resulting from the accumulation of lipids increases the production of inflammatory cytokines (IL-6, TNFα, MCP-1) and immune cells (F4-80), in addition to altering the leptin/adiponectin ratio, which is associated with metabolic dysregulation and the increased risk of chronic diseases. When the influx of fatty acids surpasses the storage capacity of adipose tissue, it leads to lipotoxicity and ectopic lipid accumulation. This accumulation promotes functional changes in the liver and pancreas. In liver tissue, lipogenesis (Srepb-1, Fas, Pparγ) is increased, whereas beta-oxidation (Cpt1-α, Pparα, Pgc1-α) is decreased, which further promotes lipid accumulation in the liver and can cause hepatic steatosis. In the pancreas, in addition to lipid accumulation, hyperglycemia caused by the diet causes pancreatic hypertrophy, with an increase in pancreatic mass (Ki-67) and insulin production, exacerbating the worsening of glucose metabolism. Therefore, the consumption of palm oil and interesterified palm oil in a high-fat diet model causes metabolic changes in the metabolism of carbohydrates and lipids, due to morpho-functional changes in adipose tissue and ectopic accumulation in other metabolic tissues (liver and pancreas).

Currently, most ultra-processed products contain palm oil or interesterified palm oil in their formulations; however, the impact of their consumption on health is unknown. In our study, we highlighted its negative effects in mice, including white adipose tissue hypertrophy, hepatic steatosis, insulin resistance, and altered gene expression of inflammatory cytokines and beta-oxidation-related genes (Fig. 6). These findings highlight the deleterious health effects of the consumption of palm oil or interesterified palm oil on the metabolism of obese mice.

## Materials and methods

### Interesterified palm oil preparation

Palm oil (PO) and interesterified (IPO) palm oils were donated by Agropalma (Limeira. São Paulo. Brazil). The industrial interesterification process implemented by Agropalma draws upon InterChem Technology. This method utilized specific operational parameters with 0.1% (w/w) sodium methoxide catalyst concentration, reaction temperatures ranging from 90 to 110 °C, a reaction duration of 20 min, reaction conducted under 30 mbar pressure using a reactor dryer with moderate agitation. The resulting interesterified fat is further refined through subsequent clarification and deodorization steps^12^.

### Regiospecific distribution analysis

High-resolution nuclear magnetic resonance spectroscopy of carbon-13 (13C NMR) of the acyl chains of triglycerides was used as a quantitative method for region-specific analyses. Samples (250 mg) were dissolved in 0.5 mL of deuterated chloroform in 5 mm NMR tubes. Spectra were obtained using a Bruker Advance DPX 300 Nuclear Magnetic Resonance (NMR) spectrometer (Silberstreifen. Rheinstetten. Germany). The 13C determination was performed at a frequency of 75.8 MHz. with a 5 mm multinuclear probe and operated at 30 °C^54^.

### Triacylglyceride composition

The triacylglyceride composition was analyzed using an Agilent 6850 Series GC System gas chromatograph (GC) using a DB17HT Agilent Catalog 122–1811 (50% phenylmethylpolysiloxane) capillary column with a length of 15 m, internal diameter of 0.25 mm, and a 0.15 µm film. Analysis conditions were as follows: split injection. ratio of 1:100; column temperature: 250 °C programmed to reach 350 °C at a rate of 5 °C/min; carrier gas, helium; a flow rate of 1.0 mL/min; injector temperature: 360 °C; detector temperature: 375 °C; injected volume: 1.0 µL; sample concentration: 100 mg/5 mL of tetrahydrofuran. The individual triacylglycerides were identified by comparing their retention times (Antoniosi Filho. Mendes. and Lanças (1995).

### Lipid classes

This was determined by size exclusion chromatography (HPSEC) on a Perkin Elmer 250 liquid chromatograph (SiconAnalytic refractive index detector; Column 1: Jordi Gel dvb 300 × 7.8 mm. 500 Å; Column 2: Jordi Gel dvb 300 × 7.8 mm. 100 Å. The analysis conditions were as follows: samples were diluted at a ratio of 1:100 (v/v) in tetrahydrofuran; mobile phase: tetrahydrofuran (HPLC grade); flow rate: 1 mL/min; injected volume: 20.0 µL.

### Animal model

The experimental protocol was approved by the Ethics Committee for Animal Experimentation of the Rio de Janeiro State University (protocol number 016/2022). All experiments were performed in accordance with the National Institutes of Health Guide for the Care and Use of Laboratory Animals (8th edition). The present study was conducted in accordance with the ARRIVE 2.0 Essential 10 guidelines (https://arriveguidelines.org). Three-month-old C57BL/6 male mice were maintained in cages under a 12-h/12-h light–dark cycle, at a mean temperature of 21 °C and with free access to food and water. The animals were randomly divided into four groups: control (C, fed a standard AIN-93M diet, 7% kcal from fat; n = 15), high-fat (HF, fat from lard), high-fat palm (HFP, fat from palm oil), and high-fat interesterified palm (HFI, fat from interesterified palm) groups. All HF groups received 50% kcal of fat (n = 15) for 8 weeks (Table 5). The palm oil (PO) and interesterified palm oil (IPO) were provided by Agropalma (Limeira, São Paulo, Brazil). IOP was produced using sodium methoxide as a catalyst through a conventional process employed in the oil and fat industry.

**Table 5:** Chemical composition and energy value of the experimental diet.

Ingredients (g/100 g)	C	HF	HFP	HFI
Corn starch (g)	46.57	23.27	23.27	23.27
Dextrinized starch (g)	15.5	11.5	11.5	11.5
Casein (g)	14.0	17.5	17.5	17.5
Sucrose (g)	10.0	10.0	10.0	10.0
Soybean (g)	4.0	4.0	4.0	4.0
Lard (g)	0	23.8	0	0
Palm oil (g)	0	0	23.8	0
Inter palm oil (g)	0	0	0	23.8
Cellulose (g)	5.0	5.0	5.0	5.0
Vitamins (g)	1.0	1.0	1.0	1.0
Minerals (g)	3.5	3.5	3.5	3.5
Energy (kcal)	400.0	519.3	519.3	519.3

The experimental diets were prepared following recommendations of the American Institute of Nutrition (IN 93M). C, control group; HF, high fat diet group; HFP, high fat palm diet group; HFI, high fat interesterified diet group.

### Food consumption, body mass and body composition

Body mass (BM) was assessed weekly by using an analytical scale (Shimadzu UX 4200H, Kyoto, Japan). Food intake was monitored thrice a week on alternate days, and consumption was measured by subtracting the remaining amount of food from the supply. Energy intake was calculated as the product of food consumption and dietary energy density. Nuclear magnetic resonance (NMR) for small living animals was performed to evaluated total fat mass. Rats were scanned using the whole-body composition analyzer NMR equipment (Bruker’s Minispec LF90 TD-NMR, Rheinstetten, Germany).

### Euthanasia and tissue extraction

At the end of the 8th week, the animals were fasted overnight and euthanized using an intraperitoneal thiopental anesthetic (200 mg/kg). Through cardiac puncture, blood samples were collected with heparinized needles and the plasma was separated by centrifugation (180×*g* at 4 °C/15 min) and stored at − 80 °C until analysis. Epididymal white adipose tissue (eWAT), liver, and pancreas were dissected and weighed on an analytical balance (Shimadzu (R) UX 4200H) and fixed in Millonig formalin (1.27 mol of formaldehyde in 0.1 M phosphate buffer, pH 7.2) for future histological sectioning or frozen at − 80 °C for molecular analysis.

### Plasma analysis

Commercial colorimetric assay kits were used to analyze the plasma levels of total cholesterol (mmol/L), triglycerides (mmol/L), alanine aminotransferase (ALT; U/L), and aspartate aminotransferase (AST; U/L) in plasma (Bioclin, Belo Horizonte, Brazil). Fasting blood glucose levels were measured using a glucometer (Accu-Chek; Roche, Germany). The plasma concentrations of insulin (EZRMI-13-K, Millipore, MO, USA), leptin (EZML-82K, Millipore, MO, USA), adiponectin (EK0596, Boster Biological Engineering Co., Ltd., Wuhan, China), interleukin 6 (IL-6, BMS603-2, Invitrogen, CA, USA), tumor necrosis factor (TNFα, #88-7324; Thermo Fisher Scientific, MA, USA), interleukin 1 beta (IL-1β #88-7013A-76, Thermo Fisher Scientific, MA, USA), glucagon-like peptide-1 (GLP-1, #BMS2194, Thermo Fisher Scientific, MA, USA) and glucose-dependent insulinotropic popypeptide (GIP, # EZRMGIP-55K, Millipore, MO, USA) were measured using mouse ELISA kits following the manufacturer's instructions.

The insulin resistance index was obtained by calculating the homeostasis model assessment index (HOMA-IR), which was determined by multiplying the insulin value (mIU/mL) by the glucose value (mmol/L) and then dividing by 22.5^55^.

### Histological, stereological, and biochemical analyses of adipose tissue, liver, and pancreas

eWAT, liver, and pancreas from the animals, which were previously fixed and later included in Paraplast Plus (Sigma-Aldrich, St. Louis, MO, USA) sliced into 5-μm-thick sections. The slides were stained with hematoxylin and eosin, and digital images of random and non-consecutive microscopic fields were obtained using an Olympus BX51 microscope coupled to a camera (Infinity 15c, Lumenera Co., Ottawa, ON, Canada) and analyzed using Image-Pro Plus software (version 7.0; Media Cybernetics, Silver Spring, MD, USA). For eWAT analysis, random fields were photographed to estimate the average cross-sectional area of adipocytes as previously described^56^. The volume density (Vv) of hepatocytes was determined using the STEPanizer software version 1.8 via 16-point tests. The results were calculated by dividing the sum of the points found by the sum of the total points of the system and are expressed as percentages^57^. Islet volume density (Vv [islet]) was estimated by point counting ^23^, and islet mass (M [islet, pancreas]) was the product of Vv [islet, pancreas] and pancreas mass^58^. Moreover, we used image analysis of sections incubated with anti-glucagon (CSB-PA002654, Cusabio, 1:100) and anti-insulin (6BA88CE779, Cloud-Clone Corp., 1:100) antibodies to estimate the alpha and beta cell volume densities (Vv [alpha-cell, islet] and Vv [beta-cell, islet]). The material was incubated with biotinylated secondary antibodies and streptavidin-peroxidase conjugates, washed with PBS, stained with liquid diaminobenzidine (DAB; Histostain Plus Kit, Invitrogen, CA, USA), and counterstained with hematoxylin. Briefly, the islet was outlined, and the image obtained in the color deconvolution window was the DAB image measured in intensity units, then converted to the optical density (ImageJ v. 1.53/FIJI http://imagej.nih.gov/ij). Finally, alpha- and beta-cell masses were estimated as the product of [Vv [alpha-cell] (or Vv [beta-cell]) and M [islets]^59^.

### Inflammatory markers in eWAT

The degree of macrophage infiltration in eWAT was assessed using immunohistochemical analysis. After deparaffinization of 5-μm-thick slices of eWAT, antigen retrieval was performed by incubation using citrate buffer (pH 6.0) for 30 min at 60 °C. Endogenous peroxidase activity was blocked using 0.3% H_2_O_2_ in PBS. Nonspecific binding of polyclonal antibodies was blocked by incubation with 5% (w/v) BSA in PBS. Subsequently, the sections were incubated with an F4/80 monoclonal antibody (sc-377009, Santa Cruz Biotechnology, Santa Cruz, CA, USA, 1:100 dilution) and amplified using a biotin–streptavidin system (Universal LSAB + kit, peroxidase; cat. no. K0679; DakoCytomation). Immunoreactive products were visualized using diaminobenzidine reagent (cat. no. K3466; Dako Cytomation) and counterstained with hematoxylin.

To evaluate lipid peroxidation, 150 µL of the sample was used in 300 µL of trichloroacetic acid (TCA). The samples were centrifuged for 20 min at 2000 rpm at 4 °C. The supernatant was separated into a microtube and supplemented with 150 µL of TBA (0.67%). The microtubes were placed in a dry bath (100 °C) for 30 min. The solution was left to cool for 5 min and subjected to spectrophotometry at 535 nm. MDA reacts with TBA to generate a pinkish product, which was read on a spectrophotometer (532 nm).

### Gene expression (mRNA quantification)

Total mRNA was extracted from the eWAT and liver (30-mg aliquots) using TRIzol reagent (Invitrogen) following the manufacturer's instructions. The RNA concentration was determined by spectrometry using BioDrop μLITE (BioDrop, UK). RNA was converted to complementary DNA (cDNA) using a reverse transcriptase enzyme (Applied Biosystems High-Capacity RNA-to-DNA Kit, Life Technologies, USA). The primers used for cDNA replication were specific to each gene to be analyzed (TaqMan Gene Expression Assay, Applied Biosystems, USA). Quantitative PCR was performed using a 7500 Fast Real-Time PCR System (Applied Biosystems, USA), and the mRNA fold change was calculated using the 2^−ΔΔCT^ method^60^. The expression of the following genes in the eWAT was evaluated: *Mcp-1* (Mm03975734_m1), *Il-6* (Mm00446190_m1), and *Tnf-α* (Mm01717107_m1). In addition, the expression of the following genes was evaluated in the liver: *Fas* (Mm 00662319_m1), *Srebp-1* (Mm00550338_m1), *Pparα* (Mm00440939_m1), *Pparγ* (Mm00440940_m1), *Pgc1α* (Mm01208835_m1) and *Cpt1α* (Mm 01231183_m1).

### Statistical analysis

Results are expressed as means ± standard deviations. Brown Frosythe and Welch's one-way ANOVA with Dunnett T3 was used to compare the four groups. All analyses were performed using GraphPad Prism version 10.2.1. Differences were considered significant at *P* < 0.05.

## Data Availability

The data used in this study are available upon reasonable request from JBD (beltrame@uerj.br).
